# The role of circular RNA HECTD1 expression in disease risk, disease severity, inflammation, and recurrence of acute ischemic stroke

**DOI:** 10.1002/jcla.22954

**Published:** 2019-07-01

**Authors:** Xiapei Peng, Ping Jing, Juan Chen, Liwen Xu

**Affiliations:** ^1^ Department of Neurology, The Central Hospital of Wuhan, Tongji Medical College Huazhong University of Science and Technology Wuhan China

**Keywords:** acute ischemic stroke, circRNA HECTD1, inflammation, recurrence, severity

## Abstract

**Background:**

This study aimed to explore the correlation of circular RNA HECT domain E3 ubiquitin protein ligase 1 (circRNA HECTD1) expression with disease risk, disease severity, inflammation, and recurrence of acute ischemic stroke (AIS).

**Methods:**

A total of 160 initial AIS patients and 160 controls were enrolled in this study. Peripheral blood mononuclear cells of AIS patients and controls were separated from blood samples to detect circRNA HECTD1 expression by RT‐qPCR. Inflammatory cytokines in serum of AIS patients were measured by ELISA. Furthermore, the National Institutes of Health Stroke Scale (NIHSS) score was used to evaluate disease severity of AIS patients. Additionally, disease recurrence was documented during follow‐up, and recurrence‐free survival (RFS) was calculated.

**Results:**

CircRNA HECTD1 expression was higher in AIS patients than that in controls, and the receiver operating characteristic (ROC) curve revealed that circRNA HECTD1 expression was of a good value in distinguishing AIS patients from controls with area under the curve (AUC) of 0.814 (95% CI: 0.768‐0.859). In AIS patients, circRNA HECTD1 expression was positively correlated with NIHSS score, CRP, and pro‐inflammatory cytokines. CircRNA HECTD1 expression was increased in AIS recurrence patients compared to non‐recurrence patients, and further, ROC curve analysis disclosed that circRNA HECTD1 expression predicted higher risk of AIS recurrence (AUC: 0.694, 95% CI: 0.586‐0.801). Additionally, circRNA HECTD1 expression was negatively correlated with RFS.

**Conclusions:**

CircRNA HECTD1 expression correlates with higher disease risk, disease severity, inflammation, and recurrence of AIS.

## 1 INTRODUCTION

Stroke is a major cause of long‐term disability and the fourth leading cause of death worldwide.^1^ Acute ischemic stroke, triggered by intracranial artery occlusion or extracranial cervical artery occlusion, accounts for around 85% of total strokes.^2^, ^3^ AIS‐caused brain tissue death, focal neurological deficits, and disability bring in great burdens to both the patients and society.^3^ Medical imaging such as computed tomography (CT), computed tomographic angiography, and magnetic resonance imaging are used for AIS treatment guidance with good accuracy.^4^ However, CT, the most commonly used one for AIS, is not sensitive to early ischemic change in brain.^2^ Additionally, the time taken to acquire, process, and interpret medical imaging data delays treatment decisions, resulting in unsatisfying prognosis. Therefore, the exploration of easily accessible and rapidly measurable biomarkers with the potentials for disease monitoring and disease management in AIS patients in clinical practice is of great importance.

Circular RNAs (circRNAs), a new class of endogenous non‐coding RNAs, are generated by back‐splicing process in eukaryotes.^5^ CircRNAs form a covalently closed loop without 5′ and 3′ end, which are often expressed in tissue‐specific or disease‐specific patterns.^5^, ^6^ Additionally, circRNAs are highly stable and abundant in blood; thus, it is easily detected in blood samples.^5^ Circular RNA HECT domain E3 ubiquitin protein ligase 1 (circRNA HECTD1) is derived from exons 23 and 24 of the HECTD1 gene.^6^ Recent studies have exhibited the role of circRNA HECTD1 in the development and progression of several diseases such as AIS and silicosis.^7^, ^8^ For example, a study discloses that knockdown of circRNA HECTD1 attenuates infract size and decreases neurological deficits in cerebral ischemia transient middle cerebral artery occlusion mouse (tMCAO) stroke models.^7^ Besides that, a preliminary study based on primary cultures of alveolar macrophages from silicosis patients demonstrates that circRNA HECTD1 involves in SiO_2_‐mediated inflammation cascade.^8^ Based on the role of circRNA HECTD1 in enlarging stroke infarct size, accelerating neurological deficits, and inflammation from previous studies, we hypothesized that circRNA HECTD1 may be involved in the development and progression of AIS. However, no clinical studies have been done in evaluating the role of circRNA HECTD1 in AIS until now. Therefore, this study initially explored the correlation of circRNA HECTD1 expression with disease risk, disease severity, inflammation, and recurrence of AIS.

## 2 MATERIALS AND METHODS

### 2.1 Patients and controls

In this study, a total of 160 initial AIS patients and 160 controls were enrolled at The Central Hospital of Wuhan, Tongji Medical College, Huazhong University of Science and Technology from January 2015 to December 2017. Inclusion criteria of AIS patients were as follows: (a) diagnosed as AIS in accordance with the Guidelines for the Early Management of Patients with AIS^9^; (b) no intracranial hemorrhage confirmed by diffusion‐weighted imaging (DWI); (c) hospitalization within 24 hours of the symptom onset; (d) age above 18 years; and (e) regular follow‐up was performable, which was evaluated by the investigator. Patients were excluded if they (a) had a history of stroke, (b) died within 24 hours after hospitalization, (c) received immunosuppressant within 4 weeks, (d) presented with active infection, (e) complicated with hematological diseases or malignancies, and (f) were breast feeding or pregnant. All controls were screened from the high‐stroke‐risk population, which was defined as subjects with at least three of following risk factors: (a) hypertension (HT), (b) atrial fibrillation (AF) or valvulopathy (val.), (c) tobacco use, (d) hyperlipidemia (HLP), (e) diabetes, (f) lack of physical exercise (PE), (g) overweight or obesity, and (h) family history of stroke. In addition, controls who had a history of stroke or malignancies were excluded.

### 2.2 Ethics statement

This study was performed according to the guidelines of the Helsinki Declaration. Ethical approval was obtained from the Institutional Review Board of The Central Hospital of Wuhan, Tongji Medical College, Huazhong University of Science and Technology. The written informed consents were collected from all participants or their guardians before enrollment.

### 2.3 Data collection

Clinical features including age, gender, body mass index (BMI), smoke, hypertension, diabetes mellitus, hyperlipidemia, hyperuricemia, and chronic kidney disease (CKD) were collected from AIS patients and controls after recruitment. In addition, for AIS patients, C‐reactive protein (CRP) level measured on the day of admission was also recorded, and the National Institutes of Health Stroke Scale (NIHSS) score evaluated for the disease severity within 24 hours of hospital admission was documented as well.

### 2.4 Sample collection

Peripheral blood samples were collected from all participants on the day of enrollment. After collection, samples were divided into two parts: One was used to separate peripheral blood mononuclear cells (PBMC) by density gradient centrifugation, and the other was used for isolation of serum by centrifugation at 4000 *g*. Both the isolated PBMC and serum were then stored at −80°C until subsequent analysis.

### 2.5 Detection of circRNA HECTD1

The expression of circRNA HECTD1 was evaluated by real‐time quantitative polymerase chain reaction (RT‐qPCR) assay. Firstly, total RNA was extracted from PBMC by using TRIzol^™^ Reagent (Invitrogen). After the extraction of total RNA, linear RNA was digested. Then, cDNA was synthesized by using PrimeScript^™^ RT reagent Kit (Perfect Real Time; Takara). Subsequently, qPCR was performed by using SYBR^®^ Premix DimerEraser^™^ (Takara). The relative expression of circRNA HECTD1 was calculated by 2^−△△Ct^ with GAPDH as internal references. Primers applied to qPCR were as follows: CircRNA HECTD1, forward (5′‐>3′): GGCAGCAGAGGATATTATCAATTAC, reverse (5′‐>3′): TTCTCGTTCGCTCCACAGT; GAPDH, forward (5′‐>3′): TGACCACAGTCCATGCCATCAC, reverse (5′‐>3′): GCCTGCTTCACCACCTTCTTGA.

### 2.6 Detection of inflammatory cytokines

The serum concentrations of the tumor necrosis factor α (TNF‐α), interleukin‐1β (IL‐1β), IL‐6, IL‐8, IL‐10, IL‐17, and IL‐22 were measured by enzyme‐linked immunosorbent assay (ELISA) using Human ELISA kits (Abcam). All experiments were performed according to the protocols provided by the manufacturer, and the detection limits of these parameters were consistent with the manufacturer's instructions.

### 2.7 Follow‐up

Surveillance and regular follow‐up for AIS patients were performed as AIS Guidelines recommended, and the last follow‐up date was December 31, 2018, with a median follow‐up duration of 25.0 months (range: 1.0‐47.0 months). Disease recurrence was documented during the follow‐up, according to that recurrence‐free survival (RFS) was calculated, which was defined as the time interval from hospital admission to the disease recurrence or death.

### 2.8 Statistical analysis

Data were displayed as mean ± standard deviation (SD), median, and interquartile range (IQR) or number (percentage). Continuous data were first determined by the Kolmogorov‐Smirnov test for normality, and depending on the distribution of the data, the parametric Student's *t* test or nonparametric Wilcoxon rank sum test was used to compare the difference between groups. Nominal categorical data between two groups were determined using the chi‐square test. Correlations analyses were performed by Spearman's rank correlation test. Diagnostic performance of variable was analyzed by receiver operating characteristic (ROC) curve, the area under the curve (AUC) as well as 95% confidence interval (CI); meanwhile, sensitivity and specificity for median value of variable were assessed as well. RFS was demonstrated by the Kaplan‐Meier curve and was determined by the log‐rank test. *P* value < 0.05 indicated a significant difference. All data analyses were performed by SPSS 22.0 statistical software (SPSS Inc.), and all figures were plotted using GraphPad Prism 7.02 software (GraphPad Software Inc.).

## 3 RESULTS

### 3.1 Baseline characteristics of AIS patients and controls

No differences of age (*P* = 0.160), gender (*P* = 0.543), BMI (*P* = 0.087), smoke (*P* = 0.576), or chronic complications were observed between AIS patients and controls. In details, the mean age was 62.5 ± 12.9 years in AIS patients and 60.6 ± 10.7 years in controls. There were 109 (68.1%) males and 51 (31.9%) females in AIS patients, 114 (71.2%) males and 46 (28.8%) females in controls. The mean BMI was 24.7 ± 2.9 kg/m^2^ in AIS patients and 24.2 ± 2.8 kg/m^2^ in controls. Smoking history was found in 78 (48.8%) AIS patients and 83 (51.9%) controls. Regarding chronic complications, there were 145 (90.6%) AIS patients and 135 (84.4%) controls with hypertension, 37 (23.1%) AIS patients and 25 (15.6%) controls with diabetes mellitus, 77 (48.1%) AIS patients and 63 (39.4%) controls with hyperlipidemia, 47 (29.4%) AIS patients and 44 (27.5%) controls with hyperuricemia, and 26 (16.3%) AIS patients and 19 (11.9%) controls with chronic kidney disease. Additionally, in AIS patients, the mean NIHSS score was 8.3 ± 3.4, the median value of CRP was 35.5 (27.3‐49.4) mg/L, and the levels of inflammatory cytokines of AIS patients were listed in Table 1.

**Table 1:** Baseline characteristics of AIS patients and controls

Items	AIS patients (N = 160)	Controls (N = 160)	*P* value
Age (y), mean ± SD	62.5 ± 12.9	60.6 ± 10.7	0.160
Gender, No. (%)			
Male	109 (68.1)	114 (71.2)	0.543
Female	51 (31.9)	46 (28.8)	
BMI (kg/m^2^), mean ± SD	24.7 ± 2.9	24.2 ± 2.8	0.087
Smoke, No. (%)	78 (48.8)	83 (51.9)	0.576
Chronic complications, No. (%)			
Hypertension	145 (90.6)	135 (84.4)	0.091
Diabetes mellitus	37 (23.1)	25 (15.6)	0.155
Hyperlipidemia	77 (48.1)	63 (39.4)	0.115
Hyperuricemia	47 (29.4)	44 (27.5)	0.710
Chronic kidney disease	26 (16.3)	19 (11.9)	0.260
NIHSS score, mean ± SD	8.3 ± 3.4	‐	‐
CRP (mg/L), median (IQR)	35.5 (27.3‐49.4)	‐	‐
TNF‐α (pg/mL), median (IQR)	78.7 (56.5‐124.5)	‐	‐
IL‐1β (pg/mL), median (IQR)	6.7 (4.5‐9.2)	‐	‐
IL‐6 (pg/mL), median (IQR)	62.3 (49.9‐81.7)	‐	‐
IL‐8 (pg/mL), median (IQR)	66.8 (49.3‐97.2)	‐	‐
IL‐10 (pg/mL), median (IQR)	16.1 (11.2‐23.4)	‐	‐
IL‐17 (pg/mL), median (IQR)	128.7 (82.1‐166.1)	‐	‐
IL‐22 (pg/mL), median (IQR)	75.1 (53.6‐108.2)	‐	‐

Note

Comparisons between groups were determined by *t* test or chi‐square test. *P* value < 0.05 was considered significant.

Abbreviations: AIS, acute ischemic stroke; BMI, body mass index; CRP, C‐reactive protein; IL, interleukinIQR, interquartile range; NIHSS, National Institutes of Health stroke scale; SD, standard deviation; TNF‐α, tumor necrosis factor‐α.

### 3.2 Comparison of circRNA HECTD1 relative expression in AIS patients and controls

**Figure 1:**
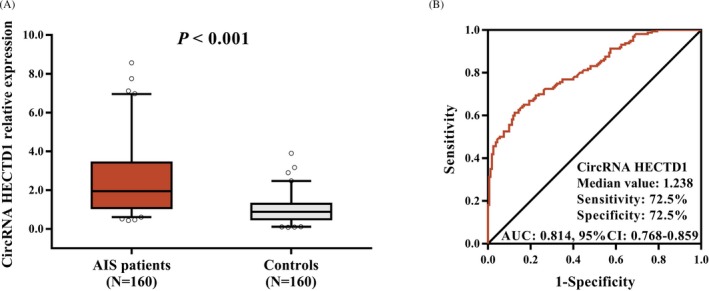
The relative expression of circRNA HECTD1 in AIS patients and controls. Increased circRNA HECTD1 in AIS patients compared to controls (A). Expression of circRNA HECTD1 exhibited a good value for differentiating AIS patients from controls (B). Comparison between AIS patients and controls was assessed by Wilcoxon rank sum test. *P* < 0.05 was considered significant. ROC curve was performed to analyze the predictive value of circRNA HECTD1 for AIS risk. CircRNA HECTD1, circular RNA HECT domain E3 ubiquitin protein ligase 1; AIS, acute ischemic stroke; AUC, area under the curve; ROC, receiver operating characteristic

CircRNA HECTD1 relative expression was higher in AIS patients (1.951 [1.078‐3.423]) than that in controls (0.886 [0.497‐1.296]) (*P* < 0.001; Figure 1A). Subsequently, ROC curve analysis disclosed that circRNA HECTD1 relative expression could differentiate AIS patients from controls (AUC: 0.814, 95% CI: 0.768‐0.859). Besides, the sensitivity and specificity were 72.5% and 72.5%, respectively, at the median value of circRNA HECTD1 in all participants (1.238; Figure 1B).

### 3.3 Correlation of circRNA HECTD1 relative expression with NIHSS score in AIS patients

**Figure 2:**
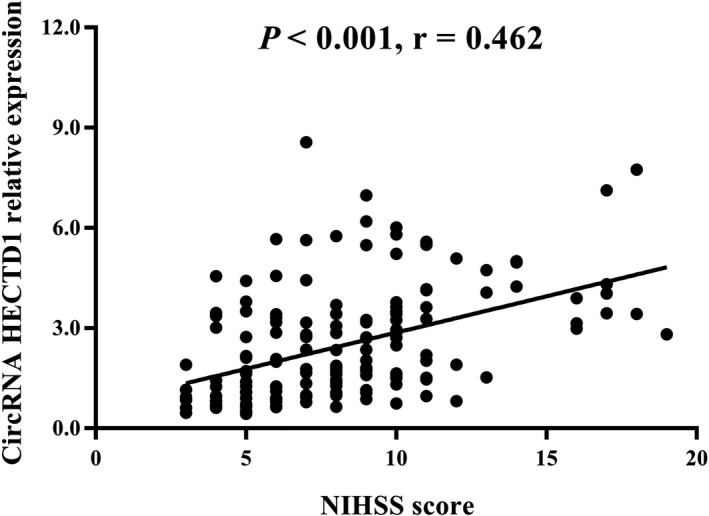
Positive correlation between circRNA HECTD1 and NIHSS score in AIS patients. CircRNA HECTD1 expression was positively associated with NIHSS score in AIS patients. Spearman's rank correlation test was used to evaluate the association of circRNA HECTD1 with NIHSS score in AIS patients. *P* < 0.05 was considered significant. CircRNA HECTD1, circular RNA HECT domain E3 ubiquitin protein ligase 1; AIS, acute ischemic stroke; NIHSS, National Institutes of Health Stroke Scale

NIHSS was used to evaluate the disease severity of AIS patients, and circRNA HECTD1 was shown to be positively correlated with NIHSS score in AIS patients (*P < *0.001, *r* = 0.462, Figure 2).

### 3.4 Correlation of circRNA HECTD1 relative expression with CRP and inflammatory cytokines in AIS patients

CircRNA HECTD1 relative expression was positively associated with CRP (*P* < 0.001, *r* = 0.388), TNF‐α (*P* = 0.002, *r* = 0.246), IL‐6 (*P* < 0.001, *r* = 0.303), IL‐8 (*P* = 0.027, *r* = 0.175), and IL‐22 (*P* = 0.001, *r* = 0.254), while circRNA HECTD1 relative expression was negatively associated with IL‐10 (*P* < 0.001, *r* = −0.298). However, no correlation of circRNA HECTD1 relative expression with IL‐1β (*P* = 0.206) and IL‐17 (*P* = 0.232) was observed in AIS patients (Table 2).

**Table 2:** Correlation of circRNA HECTD1 relative expression with inflammatory indexes and cytokines

Items	CircRNA HECTD1 relative expression	
	*P* value	Correlation coefficient (*r*)
CRP	<0.001	0.388
TNF‐a	0.002	0.246
IL‐1β	0.206	0.101
IL‐6	<0.001	0.303
IL‐8	0.027	0.175
IL‐10	<0.001	−0.298
IL‐17	0.232	0.095
IL‐22	0.001	0.254

Note

Correlation analyses were determined by Spearman's rank correlation test. *P* value < 0.05 was considered significant.

Abbreviations: CRP, C‐reactive protein; IL, interleukin; TNF‐α, tumor necrosis factor‐α.

### 3.5 Comparison of circRNA HECTD1 relative expression between recurrence patients and non‐recurrence patients

**Figure 3:**
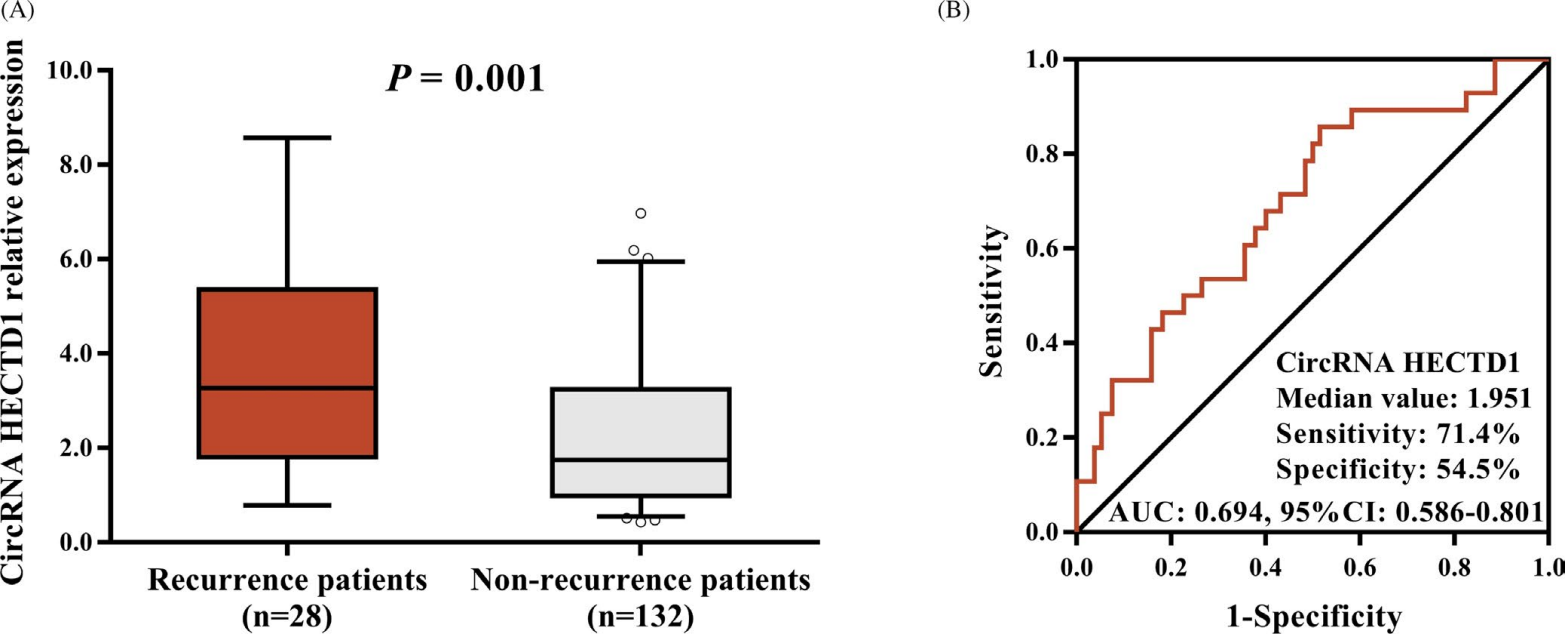
Expression of circRNA HECTD1 in recurrence patients and non‐recurrence patients. Recurrence patents had higher relative expression of circRNA HECTD1 than that in non‐recurrence patients (A). Expression of circRNA HECTD1 could differentiate recurrence patients and non‐recurrence patients (B). Comparison between recurrence patients and non‐recurrence patients was analyzed by Wilcoxon rank sum test. *P* < 0.05 was considered significant. ROC analysis was conducted to detect the ability of circRNA HECTD1 in differentiating recurrence patients from non‐recurrence patients. CircRNA HECTD1, circular RNA HECT domain E3 ubiquitin protein ligase 1; AUC, area under the curve; ROC, receiver operating characteristic

In AIS patients, 28 patients had recurrence and 132 did not have recurrence during follow‐up. Compared to non‐recurrence patients (1.747 [0.990‐3.242]), circRNA HECTD1 relative expression was elevated in recurrence patients (3.270 [1.807‐5.352]) (*P* = 0.001; Figure 3A). Further, ROC analysis showed that circRNA HECTD1 expression predicted higher risk of AIS recurrence (AUC: 0.694, 95% CI: 0.586‐0.801). The sensitivity and specificity were 71.4% and 54.5%, respectively, at median value of circRNA HECTD1 relative expression in AIS patients (1.951; Figure 3B).

### 3.6 Correlation of circRNA HECTD1 relative expression with RFS

**Figure 4:**
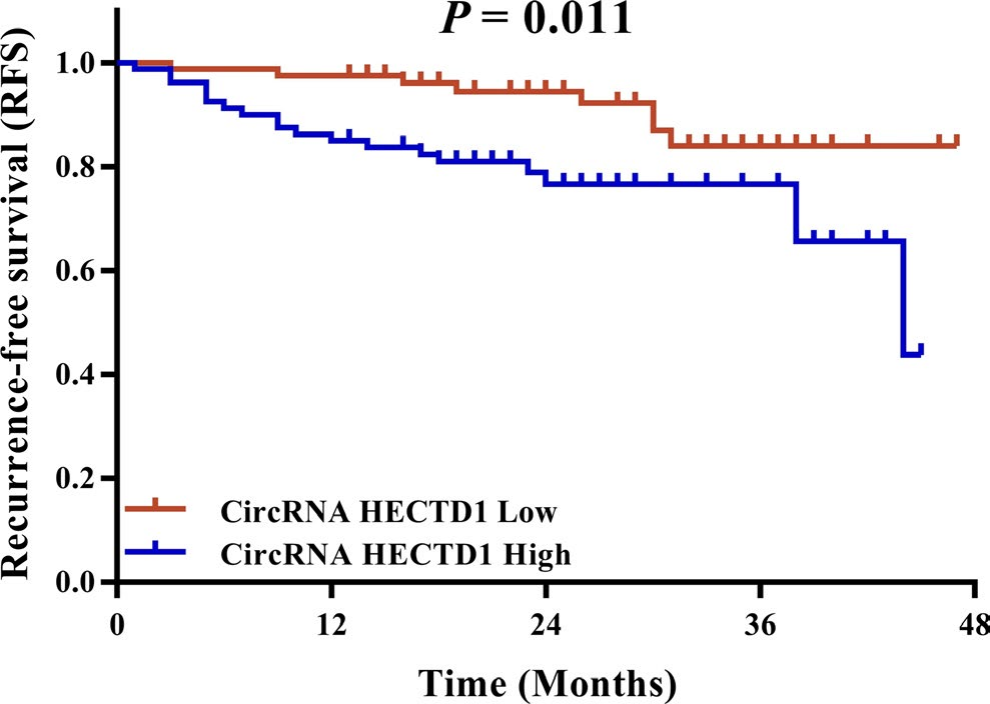
Negative correlation between circRNA HECTD1 expression and RFS. RFS was decreased in patients with circRNA HECTD1 high expression compared to patients with circRNA HECTD1 low expression. Kaplan‐Meier curve and log‐rank test were used to compare RFS between patients with circRNA HECTD1 high expression and patients with circRNA HECTD1 low expression. *P* < 0.05 was considered significant. CircRNA HECTD1, circular RNA HECT domain E3 ubiquitin protein ligase 1; RFS, recurrence‐free survival

AIS patients were divided into two categories: patients with circRNA HECTD1 low expression and patients with circRNA HECTD1 high expression based on their baseline median value of circRNA HECTD1 (1.951). Subsequently, K‐M survival analysis exhibited that patients with circRNA HECTD1 high expression presented with shorter RFS (mean: 36.1 months, 95% CI: 32.7‐39.5 months) compared to patients with circRNA HECTD1 low expression (mean: 43.2 months, 95% CI: 40.8‐45.7 months; *P* = 0.011; Figure 4).

## 4 DISCUSSION

In the present study, we discovered that: (a) CircRNA HECTD1 expression was increased in AIS patients compared to controls, and ROC curve analysis revealed that circRNA HECTD1 expression was of a good value in distinguishing AIS patients from controls; (b) CircRNA HECTD1 expression was positively associated with NIHSS score and inflammation in AIS patients; and (c) CircRNA HECTD1 expression predicted higher risk of AIS recurrence.

In recent years, the high specificity and stability of circRNAs make them suitable candidates as biomarkers; furthermore, certain circRNAs have been proposed as transcriptional and translational regulators in pathological aspects of several cardiovascular diseases.^10^ For instance, circular antisense non‐coding RNA in the INK4 locus expression is significantly higher in the whole blood from carriers of the coronary artery disease–protective haplotype compared to non‐carriers.^11^ CircRNA hsa_circ_025016 is upregulated in postoperative arterial fibrillation (PoAF) patients compared to no PoAF patients.^12^ These collective evidences suggest that circRNAs might serve as biomarkers for predicting cardiovascular disease risk. CircRNA HECTD1 is highly abundant in brain, heart, and liver.^13^ In AIS, the potential source of circRNA HECTD1 is from brain since AIS is caused by the sudden loss of blood circulation to an area of the brain.^2^ CircRNA HECTD1 has been suggested as a regulator in the pathogenesis of AIS in tMCAO stroke models.^7^ However, the role of circRNA HECTD1 has not been evaluated in cardiovascular diseases in clinical studies. Our study showed that PBMC circRNA HECTD1 expression was increased in AIS patients than that in controls, and ROC curve analysis showed that circRNA HECTD1 was of a good value in differentiating AIS patients from controls. This may be explained by the following: CircRNA HECTD1 may enlarge infarct size, aggregate neuron deficits, and trigger inflammation, resulting in a higher AIS risk.^7^, ^8^ Thus, circRNA HECTD1 might be used as a novel biomarker in predicting AIS risk.

A few reports have demonstrated that circRNA HECTD1 expression accelerates progression of several diseases such as AIS and silicosis.^7^, ^8^, ^14^ In tMCAO stroke models, circRNA HECTD1 expression is elevated in ischemic brain tissues, and the knockdown of circRNA HECTD1 markedly reduces infarct areas, attenuates neuronal deficits, and ameliorates astrocyte activation.^7^ Besides that, a recent study shows that circRNA HECTD1 expression has a role in SiO_2_‐triggered inflammatory response in silicosis patient.^8^ However, in clinical studies, no correlation of circRNA HECTD1 expression with disease progression of AIS has been investigated. Our study showed a positive correlation of circRNA HECTD1 expression with NIHSS score that evaluated disease severity in AIS patients. This result might be explained by the following: (a) CircRNA HECTD1 expression might function as microRNA (miRNA) sponge of certain miRNAs such as miRNA‐142, targeting downstream pathway to promote astrocyte activation via the regulation of autophagy and enlarge infarct area, thus leading to increased disease severity of AIS patients.^7^ (b) CircRNA HECTD1 expression might promote the expression of pro‐inflammatory cytokines, activated inflammation and immune responses, thus contributed to the onset and progression of AIS.^15^ This view is further supported by the following analysis of our study that circRNA HECTD1 was positively associated with pro‐inflammatory index and cytokines (CRP, TNF‐α, IL‐6, IL‐8, and IL‐22). These results suggested that circRNA HECTD1 might act as a pro‐inflammatory gene through regulating the expression of inflammatory index and cytokines in AIS, while the correlation coefficient of circRNA HECTD1 with pro‐inflammatory index and cytokines was below 0.3, which meant the correlation of circRNA HECTD1 with pro‐inflammatory index and cytokines was weak. The possible reasons were as follows: (a) There were outlier data regarding circRNA HECTD1 expression in AIS patients, which decreased the overall correlation coefficient. (b) CircRNA HECTD1 expression might also mediate and trigger other pathways to accelerate disease severity of AIS; thus, the correlation of circ‐HECTD1 with inflammatory markers was weak.

The recurrence of AIS is still a troublesome issue to be solved in clinical practice because the rate of AIS recurrence is as high as 7%‐20% at 1 year and 16%‐35% at 5 years, which is a strong contributor of additional disability, mortality, and greater cognitive decline.^16^, ^17^ Currently, no study has been done to investigate the correlation of circRNAs with AIS recurrence. Our study identified that circRNA HECTD1 expression could predict higher recurrence AIS risk. Furthermore, the RFS of AIS patients was shorter in circRNA HECTD1 high group than that in circRNA HECTD1 low group. The possible explanation of these results might be as follows: (a) Increased circRNA HECTD1 expression might promote astrocyte activation via its downstream pathway to increase infract area, resulting in increased disease severity with subsequent higher disease recurrence.^7^ (b) CircRNA HECTD1 upregulated the level of pro‐inflammatory cytokines and stimulated inflammatory and immune responses, which accelerated disease progression and subsequently increased AIS recurrence.

There were some limitations in this study: (a) The sample size was relatively small, and a larger sample is necessary for further validation. (b) The circRNA HECTD1 expression was only measured on the day of admission; however, as a disease‐monitoring biomarker, the difference of circRNA HECTD1 expression was not compared between the day of admission and after treatment. (c) Inflammatory cytokines were not assessed in the controls, and this should be verified in the future study. (d) Only one scale (NIHSS score) was used to evaluate disease severity, and more motion and cognitive‐related assessments are needed in the future. (e) The detailed mechanism of circRNA HECTD1 in regulating the development of AIS was not explored in this study. Therefore, further in vivo and in vitro experiments in investigating the detailed mechanism of circRNA HECTD1 in AIS should be carried out in the future.

In conclusion, circRNA HECTD1 expression correlates with increased disease risk, disease severity, elevated inflammation, and recurrence risk of AIS, which discloses the potential of circRNA HECTD1 as a biomarker for AIS.

## CONFLICTS OF INTEREST

The authors declare that they have no conflicts of interest.
